# Serum Phosphorylated Neurofilament-Heavy Chain, a Potential Biomarker, is Associated With Peripheral Neuropathy in Patients With Type 2 Diabetes

**DOI:** 10.1097/MD.0000000000001908

**Published:** 2015-11-06

**Authors:** Xiaona Qiao, Shuo Zhang, Weiwei Zhao, Hongying Ye, Yehong Yang, Zhaoyun Zhang, Qing Miao, Renming Hu, Yiming Li, Bin Lu

**Affiliations:** From the Department of Endocrinology and Metabolism, Huashan Hospital, Fudan University, Shanghai, China.

## Abstract

Neurofilament (NF), one of the major axonal cytoskeletal proteins, plays a critical role in degenerative diseases in both the central and the peripheral nervous systems. The aim of this study is to explore the relationship between serum phosphorylated neurofilament-heavy chain (pNF-H) and diabetic peripheral neuropathy (DPN) in patients with type 2 diabetes.

Serum pNF-H concentrations were measured by ELISA in hospitalized patients with and without DPN (n = 118). DPN was assessed by clinical symptoms, signs, and electromyography.

Compared with the non-DPN group (311.98 [189.59–634.12] pg/mL), the confirmed group (605.99 [281.17–1332.78] pg/mL) patients had the higher serum pNF-H levels (*P* = 0.007). DPN was significantly correlated with C-peptide (*r* = −0.269), total cholesterol (TC) (*r* = 0.185), and pNF-H (*r* = 0.258). Serum pNF-H levels were independently associated with DPN (*P* = 0.004), even after adjusting for age, sex, duration of diabetes, fasting plasma glucose, glycosylated hemoglobin A_1c_, TC, C-peptide, urinary albuminto/creatinine ratio, and estimated glomerular filtration rate. Compared with pNF-H quartile 1 (referent), patients in quartile 3 (odds ratio [OR], 3.977; 95% confidence interval [CI], 1.243–12.728; *P* = 0.021) and quartile 4 (OR, 10.488; 95% CI, 3.020–34.429; *P* = 0.000) had the higher risk of DPN after adjusting for the confounders.

Serum pNF-H levels might be associated with the DPN, and the correlationship between serum pNF-H and DPN should be further studied.

## INTRODUCTION

Diabetic peripheral neuropathy (DPN) is one of the most common chronic complications of diabetes mellitus. With a prevalence ranging from 10% to 70% and the high risk of foot ulcers and nontraumatic amputations, DPN has become one of the strongest determinants of reduced health-related quality of life in patients with type 2 diabetes mellitus.^1–4^ Early detection and good glycemic control have been proven to either prevent or delay adverse outcomes associated with DPN.^5,6^

In comparison to the prediction of late-stage complications such as ulceration, amputation, and death, few studies on the prediction of future onsets of DPN were performed.^7–9^ One study showed that individual nerve conduction (NC) study parameters or their simple combinations were valid measures for identification and future prediction of DPN^7^; however, the nerve electrophysiological examination was not routinely recommended for predicting or diagnosing DPN,^10^ and only sometimes used for the exclusion of other conditions.^11^ Subclinical DPN could be diagnosed only by NC criteria, but small-fiber polyneuropathies were always plagued with missed diagnosis by nerve electrophysiological examinations due to the insensitivity for detecting small-fiber neuropathy.^12^ The neurological examination and symptoms are relatively subjective. To date, these predictive and diagnostic tools have some limitations, so the biomarker specific to neural damage, as an easy and noninvasive test, is still searched.^13^

Neurofilaments (NFs), which is one of the predominant structural proteins in axons, enact important functions in both the establishment and sustainment of neuronal tensile strength, integrity of axons, and, possibly, intracellular transport guidance to axons and dendrites. Exclusively expressed in neurons, NFs act together with other cytoskeleton proteins, such as microtubules and microfilament, to form and maintain cell shape and contribute to the delivery of particles and organelles within the cytoplasm. NFs consist of 3 subunits, namely NF-light (L), NF-medium (M), and NF-heavy (H), as defined by their molecular weight.^14^ NF-L is fundamental for the correct assembly of NFs and maintenance of axonal caliber. NF-M plays a role in the formation of cross-bridges, stabilization of the filament network and longitudinal extension; NF-H also participates in the formation of cross-bridges and interacts with microtubules, microfilament, and other cytoskeletal elements. The function of NFs appears to be closely related to their phosphorylation status. The phosphorylation of NF-H and NF-M is believed to be involved in regulating interfilament spacing and axonal caliber.^15^ Phosphorylated NF-H (pNF-H) mediates interactions with other cytoskeletal elements, particularly microtubules.^16^ Abnormally phosphorylated NFs in the cell bodies have been proposed to be the common feature of neurodegenerative diseases, such as amyotrophic lateral sclerosis and Alzheimer disease.^16^ Of the various NFs, pNF-H is relatively resistant to proteolysis,^17^ so it was less likely to be degraded in blood than the other NFs. Because of the stability and testability of pNF-H, most studies focus on the potential value of the biomarker in neurodegenerative diseases.^14,18,19^ Therefore, we hypothesize that serum pNF-H levels may be a potential biomarker for DPN.

## MATERIALS AND METHODS

### Sample Size Estimation

In the pilot experiment, pNF-H levels were measured in 62 patients (34 with non-DPN and 28 with DPN). We assumed a 2-sided α error of 5%, and a statistical power of 80% to determine that a total of 57 patients were needed per group. Finally, the 118 patients in our study were consecutively enrolled.

### Study Population

From March 2011 to August 2012, 118 hospitalized patients at Huashan Hospital with type 2 diabetes were included in this study. Diabetes status was biochemically confirmed according to the World Health Organization diagnostic criteria for the classification of diabetes.^20^ All subjects provided informed consent, and the study was approved by the Ethics committee of Huashan Hospital of Fudan University. Patients with alcohol abuse, vitamin deficiency, renal dysfunction (glomerular filtration rate [GFR] <60 mL/min/1.73 m^2^), acute cerebral infarction, amyotrophic lateral sclerosis, Alzheimer disease, Parkinson disease, and other disorders of the central nervous system were excluded from this study.

### Clinical Feature Measurement

Height and weight were assessed with the patients in light clothing and without shoes. Body mass index (BMI) was calculated as body weight (in kg) divided by the square of the height (in meters). The blood pressure of each seated subject was obtained after 10 minutes of rest using a mercury sphygmomanometer. Three consecutive readings with 1-minute intervals were averaged as the blood pressure.

### Laboratory Parameter and pNF-H Measurements

All included patients were fasted overnight before collecting blood samples. Serum pNF-H levels were analyzed using an ELISA kit (Human Phosphorylated Neurofilament H; BioVendor Research and Diagnostic Product, Heidelberg, Germany). The lower limit of detection for pNF-H was 23.5 pg/mL. Glycosylated hemoglobin A_1c_ (HbA_1c_), glycated albumin, and C-peptide were determined by high-pressure liquid chromatography, liquid enzymatic assay, and radioimmunoassay, respectively. Fasting plasma glucose (FPG), total cholesterol (TC), triglyceride, high-density lipoprotein-cholesterol, low-density lipoprotein-cholesterol, and serum creatinine were analyzed using an automatic analyzer (AU640; Olympus, Shinjuku, Japan). Urinary creatinine levels were determined using the alkaline picrate method. Urinary albuminto/creatinine ratio (ACR) was calculated as albumin (mg) divided by creatinine (g) and 3 times of ACRs were averaged. The GFR was estimated by the following Modification of Diet in Renal Disease equation: estimated glomerular filtration rate (eGFR) (mL/min/1.73 m^2^) = 170 × serum creatinine (mL/dL)^−0.999^ × age (year)^−0.176^ × serum urea nitrogen (mg/dL)^−0.17^ × serum albumin (g/dL)^0.318^ × 0.742 (in female patients).

### Neurological Symptoms and Physical Examinations

Each patient's medical records were reviewed regarding neurological symptoms and neurological physical examinations. Neuropathy symptom score and the neuropathy disability scores were calculated.^1^ Symptoms like burning, numbness, tingling, fatigue, cramping, or pain in the legs were taken into account. Pinprick, temperature, vibration perception, and ankle reflex were also tested.^1^

### NC Velocity Tests

Conventional sensory and motor NC studies were performed by a single neurologist. All nerve stimulations, including those of the median, ulnar, common peroneal, and tibial motor nerves, and the median, ulnar, and superficial peroneal and of the sural sensory nerves in both limbs, were performed with a Keypoint 4 electromyography (EMG) machine (Keypoint 4; Tonsbakken 16-18, DK-2740 Skovlunde, Denmark). Local skin temperature was maintained at 32°C to 33°C. The variables were considered abnormal when they exceeded the mean ± 2 SD that were established in the authors’ laboratory. A decrease in NC velocity (NCV) was assumed if the conduction velocity was <50.0 m/s in the median motor nerve, <51.0 m/s in the ulnar motor nerve, <39.4 m/s in the tibial motor nerve, <39.8 m/s in the common peroneal motor nerve, and <50.8 m/s in the median sensory nerve, <51.6 m/s in the ulnar sensory nerve, <41.3 m/s in the superficial peroneal nerve, <41.9 m/s in the sural nerve. When ≥2 nerves were tested abnormal, NC was identified as abnormal.^21^

### Diagnosis of DPN

DPN was categorized into 3 levels, according to clinical findings and NC results based on the modified Toronto Expert Consensus^12^: non-DPN, in which patients had neither clinically evident DPN nor abnormal NC tests; clinically evident DPN where patients had at least 2 positive results among sensory symptoms, signs, or reflex abnormalities in accordance with a distal symmetrical polyneuropathy, and with the normal NC studies; and confirmed DPN, in which patients had at least one abnormal nerve parameter (of NCV, amplitude, latency, and F-wave) in ≥2 nerves among the median, peroneal, and sural nerves,^22^ with or without clinical signs and symptoms. The DPN (if not specified) consisted of clinically evident and confirmed DPN.

### Statistical Analysis

General demographic and laboratory characteristics were summarized as mean ± SD or median (25th–75th percentiles). Levene test was used to assess variance homogeneity. The least significant difference and Dunnett test were used to compare differences between groups with continuous variables, and χ^2^ analyses were used to assess differences between categorical variables. The Kruskal–Wallis test was used to compare the serum pNF-H among the 3 diabetes groups. Spearman correlation was used to examine the relationships between DPN and clinical characteristics. Patients were also categorized into quartiles based on the serum pNF-H level: quartile 1, pNF-H <235.03 pg/mL; quartile 2, 235.03 pg/mL ≤ pNF-H ≤366.2 pg/mL; quartile 3, 366.22 pg/mL ≤ pNF-H ≤776.92 pg/mL; quartile 4, pNF-H ≥776.93 pg/mL. Logistic regression analysis was used to evaluate the risk of DPN in different pNF-H quartiles. Analyses were performed using SPSS 17.0 (IBM Corporation, Somers, NY).

## RESULTS

The demographic and laboratory data for the patients with and without DPN have been summarized in Table 1. No significant differences were identified between the groups in terms of age, sex, blood pressure, and BMI. Compared with the clinically evident DPN and non-DPN groups, the confirmed DPN patients were with significantly longer duration, higher HbA_1c_, and lower C-peptide. The rate of antidiabetic drugs (ie, metformin, sulfonylureas, insulin) and antihypertension drugs (Angiotensin-Converting Enzyme Inhibitors or Angiotensin Receptor Blocker) among the 3 groups were not significantly different.

**TABLE 1:**
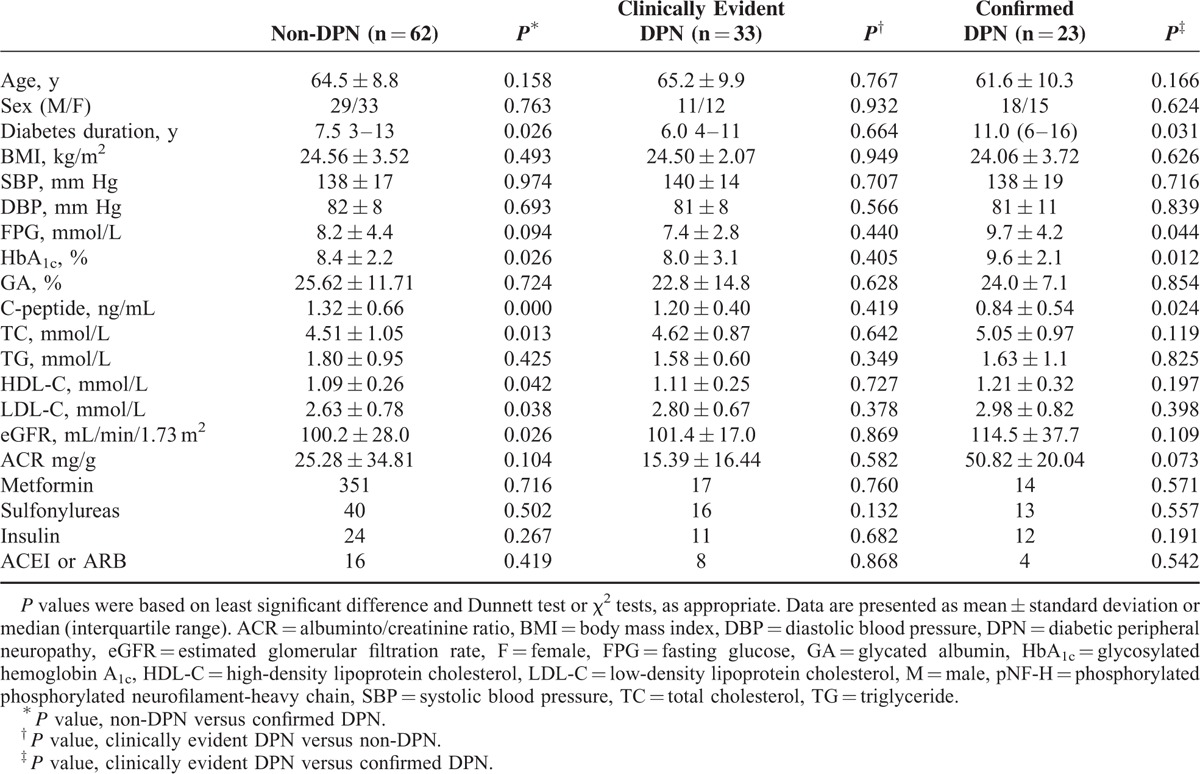
Characteristics of Subjects

**FIGURE 1:**
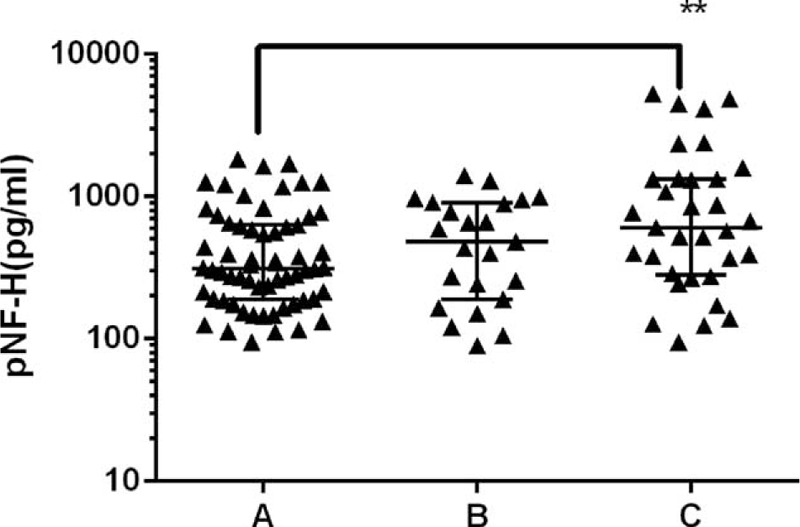
Serum pNF-H levels in groups of non-DPN, clinically evident DPN, and confirmed DPN, which are referred to as A, B, and C, respectively. Each dot represents serum pNF-H concentration in a single individual. Median and interquartile ranges have been indicated in plots by solid lines. Statistical analysis revealed significantly increased serum pNF-H levels in the confirmed DPN group compared with the non-DPN group (*P* = 0.007). DPN = diabetic peripheral neuropathy, pNF-H = phosphorylated neurofilament-heavy chain.

Compared with the patients without DPN (311.98 [189.59–634.12] pg/mL), the confirmed DPN patients (605.99 [281.17–1332.78] pg/mL) possessed higher pNF-H levels (*P* = 0.007). A higher trend was also seen when compared with a clinically evident DPN group (479.22 [188.49–909.18] pg/mL); however, that difference was not significant (*P* = 0.154) as shown in Figure 1.

DPN (both the clinically evident and confirmed groups) was significantly correlated with C-peptide (*r* = −0.269), TC (*r* = 0.185), and pNF-H (*r* = 0.258) as shown in Table 2. As shown in Table 3, after adjustment for age, sex, duration of diabetes, FPG, HbA_1c_, TC, C-peptide, ACR, and eGFR, the serum pNF-H levels remained independently associated with DPN (*P* = 0.005) (Table 3).

**TABLE 2:**
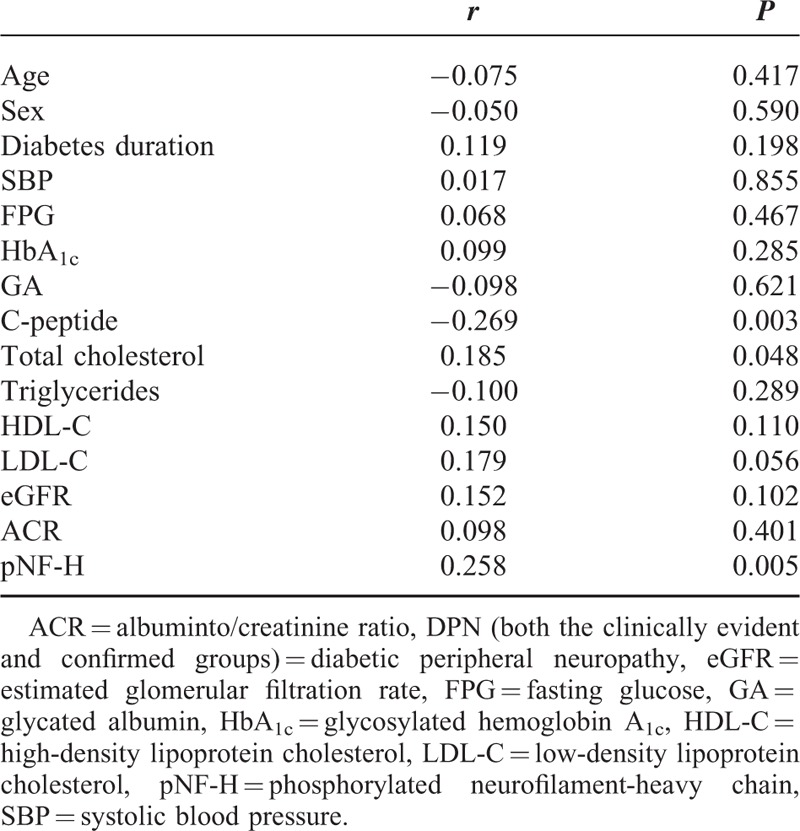
Spearman Correlation Analysis Between DPN and Clinical Characteristics

**TABLE 3:**
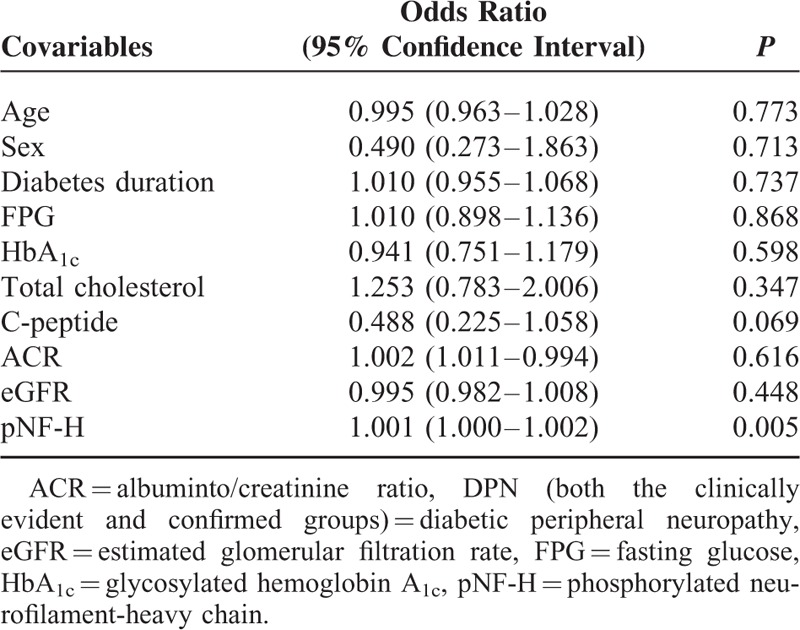
Multiple Regression Analysis of the Relationship Between pNF-H and DPN

Logistical regression analysis showed that compared with pNF-H quartile 1 (referent), patients in quartile 3 (odds ratio [OR], 3.977; 95% confidence interval [CI], 1.243–12.728; *P* = 0.021) and quartile 4 (OR, 10.488; 95% CI, 3.020–34.429; *P* = 0.000) had a higher risk of DPN, but not those in quartile 2 (OR, 1.163; 95% CI, 0.291–4.644; *P* = 0.831) after adjusting for age, sex, duration of diabetes, FPG, HbA_1c_, TC, C-peptide, ACR, and eGFR. However, the trend test analysis showed that the statistical difference of prevalent DPN among quartile 2, quartile 3, and quartile 4 referred to quartile 1 (*P* < 0.001), as shown in Table 4.

**TABLE 4:**
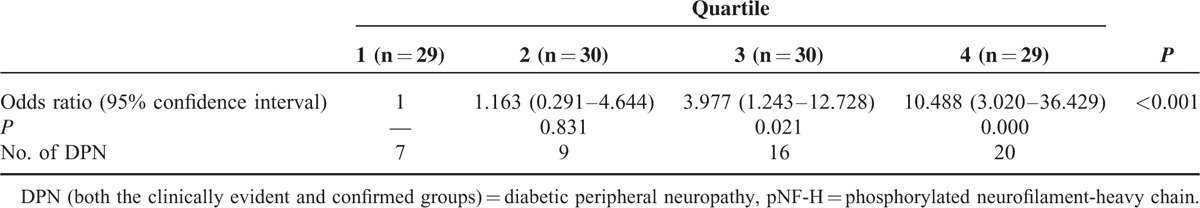
DPN Risk in Different pNF-H Quartiles

## DISCUSSION

This study supports the hypothesis that a relationship between serum pNF-H levels and DPN exists. Serum pNF-H levels in DPN patients were much higher than in non-DPN patients, and the elevated serum pNF-H levels were associated with DPN independent of multiple covariables.

Surrogate markers of diabetic neuropathy are actively being sought to facilitate the diagnosis, measure the progression, and assess the benefits of therapeutic intervention in patients with DPN. For example, Ybarra et al^23^ reported that transforming growth factor-beta 1 (TGFβ1) was significantly higher in patients with DPN than in those without, and indicated that TGFβ1 is as an important biomarker molecule for DPN screening. Another study found that serum neuron-specific enolase (NSE) levels elevated slightly in diabetic patients compared with those without diabetes and that NSE levels increased greatly in diabetic patients with neuropathy compared with those without neuropathy. The study concluded that NSE levels not only increased with but also were intimately correlated to the stages of neuropathy.^13^ Serum cystatin C (CysC) levels were also reported significantly higher in DPN patients and those elevated serum CysC levels were associated with DPN independent of covariables.^24^ Despite these positive results, none of the above-mentioned biomarkers are related directly to the structure of nerve itself, which might overlap with disorders affecting other systems, such as a generalized inflammatory state, renal insufficiency, and lung tumor.

NF, one of the major axonal cytoskeletal proteins, was related to the injury of either central, peripheral, or both nervous systems.^25–29^ Increased pNF-H levels were also observed in patients with an acute and chronic phases of neurological diseases such as multiple sclerosis, febrile seizures, hypoxic-ischemic encephalopathy, and other conditions.^30–32^ Consistent with those studies, the current study identified a significant increase in serum pNF-H levels in DPN patients after excluding central nervous system disorders and other diseases reported to cause elevated serum. This is not altogether surprising considering that diabetes causes a variety of functional and structural changes in the central and peripheral nervous system. For example, in patients with diabetes, hyperglycemia-induced formation of advanced glycation end products results in an impairment of axonal transport, a reduction in axon caliber, and a reduced capacity for nerve regeneration, which are closely related to the axonal cytoskeletal proteins such as tubulin and NFs.^33^

The reasons for the elevated serum pNF-H levels in DPN patients have not yet been established. Animal studies reported that reduced myelinated fiber sizes were correlated with loss of axonal NF in peripheral nerve of chronically streptozotocin diabetic rats.^34^ Moreover, a decreased expression and transport of NF subunits to the distal axon has been proposed as an important role in the axonal degeneration, which has been observed in diabetic animal models and humans.^15,35,36^ In the current study, the authors propose that the release of the accumulated pNF-H into the extracellular space leads to the reduced expression detectably and the elevated pNF-H level in serum, as shown herein.

The HbA_1c_ represents the mean blood concentration over the last 2 to 3 months (depending on the individual), and provides a much more stable indication of long-term glycemic control than blood and urinary glucose tests. Fasting serum C-peptide measurement is used to measure endogenous insulin reserve in diabetic patients. In general, the diabetic patients with lower C-peptide levels had the poorer blood glucose control as shown in our results. Moreover, the confirmed DPN group had the longer duration of diabetes, and the islet function should also be poorer in this group; therefore, the reduction of C-peptide levels in the confirmed DPN group was reasonable due to the poorer islet function. In addition, higher C-peptide levels might be a protective factor for DPN, which could explain the low C-peptide level in DPN patients.^37,38^

The patients with abnormality of EMG results had the higher serum pNF-H level, which is related to the function of pNF-H to maintain the integrity of the axonal cytoskeleton of nerve.^26^ pNF-H is released into serum during axonal injury in DPN patients, and Spearman's correlation analysis showed that elevated serum pNF-H levels were closely related with NCV (data not shown). Because pNF-H is concentrated in larger-diameter axons, the abnormalities of small and unmyelinated fiber in the patients with neurological clinical signs and symptoms, but normal EMG results (ie, the clinically evident DPN group), just showed mild higher serum pNF-H levels as confirmed in this study.

As with all studies, there are indeed some limitations. First, the potentially different levels of pNF-H in subcategories of DPN were not analyzed, so the relationship between severity of DPN and pNF-H level was not clarified. Second, the study did not include nonhospitalized type 2 diabetic patients, meaning that it would not be possible to directly apply these study results to patients with good glycemic control. Third, as the character of cross-sectional, this study could not evaluate the “cause and effect” relationship between elevated serum pNF-H levels and DPN. As such, larger-scale studies that include additional subcategories of DPN and longitudinal evaluation of diabetic patients without neuropathy, but high levels of pNF-H, are warranted in future.

## CONCLUSION

This study found that serum pNF-H levels might be associated with DPN. Future large-scale studies are expected to further define the association of DPN and pNF-H levels.
